# HPLC-ESI-MS/MS of Imidazole Alkaloids in *Pilocarpus microphyllus*

**DOI:** 10.3390/molecules13071518

**Published:** 2008-07-30

**Authors:** Alexandra CH F Sawaya, Ilka Nacif Abreu, Nathalia Luiza Andreazza, Marcos N. Eberlin, Paulo Mazzafera

**Affiliations:** 1 Tho*MS*on Mass Spectrometry Laboratory, Institute of Chemistry, State University of Campinas - UNICAMP, Campinas, SP, Brazil; 2 Bandeirante University of São Paulo, UNIBAN, São Paulo, SP, Brazil; 3 Department of Plant Physiology, Institute of Biology, State University of Campinas - UNICAMP, Campinas, SP, Brazil; 4 Umeå Plant Science Centre – Umeå – Sweden

**Keywords:** Jaborandi, *Pilocarpus microphyllus*, imidazole alkaloids, pilocarpine, HPLC-ESI-MS/MS.

## Abstract

Pilocarpine, an important imidazole alkaloid, is extracted from the leaves of *Pilocarpus microphyllus* (Rutaceae), known in Brazil as *jaborandi* and used mainly for the treatment of glaucoma. *Jaborandi* leaves also contain other imidazole alkaloids, whose pharmacological and physiological properties are unknown, and whose biosynthetic pathways are under investigation. In the present study, a HPLC method coupled with ESI-MS^n^ was developed for their qualitative and quantitative analysis. This method permits the chromatographic separation of the imidazole alkaloids found in extracts of jaborandi, as well as the MS/MS analysis of the individual compounds. Thus two samples: leaves of *P. microphyllus* and a paste that is left over after the industrial extraction of pilocarpine; were compared. The paste was found to contain significant amounts of pilocarpine and other imidazole alkaloids, but had a slightly different alkaloid profile than the leaf extract. The method is suitable for the routine analysis of samples containing these alkaloids, as well as for the separation and identification of known and novel alkaloids from this family, and may be applied to further studies of the biosynthetic pathway of pilocarpine in *P. microphyllus.*

## Introduction

*P. microphyllus* (Rutaceae) is originally from the Amazon region in Brazil where it is known as *jaborandi* [1]. This plant is a rich source of imidazole alkaloids, of which pilocarpine is the best known and the only one being economically exploited. Pilocarpine has been well studied as it is used not only for the treatment of glaucoma [2] but also as a stimulant of sweat and lachrymal glands [3,4]. 

The possibility of production of pilocarpine by callus cell lines in bioreactors has been evaluated, with the objective of protecting the *jaborandi* plant from uncontrolled exploitation [5]. Studies using electrospray ionization mass spectrometry (ESI-MS) to investigate the regulation of pilocarpine biosynthesis in *P. microphyllus* callus showed that pilosine (another imidazole alkaloid) was also produced depending on the cell line and treatment [6]. Due to the lack of standards of other imidazole alkaloids produced in *jaborandi*, posterior studies were conducted using electrospray ionization mass spectrometry (ESI-MS). These studies resulted in the characterization, by direct insertion tandem mass spectrometry (ESI-MS/MS and ESI-MS/MS/MS), of five known imidazole alkaloids, as well as three new alkaloids, suggesting the existence of three different biosynthetic pathways for imidazole alkaloids in the *jaborandi* plant [7]. Although the presence of pilocarpine has been reported in 10 species of the *Pilocarpus* genus [1], there is no information as to the presence and concentration of the other imidazole alkaloids, whose pharmacological properties are still unknown. 

Diverse chromatographic methods exist for the analysis of pilocarpine, mainly for the purpose of quality control of eye drops. Most of the analytical methods applied to the quantification of pilocarpine employ HPLC with either octadecyl [8,9], phenyl [10,11] or cyclodextrin [12] columns and UV detection. More recently, monolithic HPLC columns have also been tested [13]. As pilocarpine and related compounds lack chromophoric groups, UV detection must be carried out at low wavelengths (210-214 nm) where several other compounds may interfere [14], particularly when plant extracts are analyzed. Lower detection limits and richer structural information could be achieved by HPLC-ESI-MS analysis. However triethylamine, which is commonly used in buffer solutions to prevent tailing [13] is incompatible with ESI-MS detection. Merbel *et al*. [14] tested several stationary phases and eluents, determining that a combination of an Inertsil octadecyl column with an eluent using an ammonium acetate buffer and acetonitrile organic modifier was compatible for the LC-APCI-MS separation of pilocarpine, isopilocarpine and their respective acids extracted from plasma. It has also been our experience also that not all brands of octadecyl columns are capable of adequately separating imidazole alkaloids because of tailing.

Herein we describe a HPLC-ESI-MS/MS method developed for the analysis of imidazole alkaloids in the extracts of *P. microphyllus* samples. The method used is based on concepts used in previous papers, but has been modified and improved, in order to separate over a dozen imidazole alkaloids in a single 22 minute chromatographic run. The soft ionization technique used (ESI) permits the observation of protonated ([M + H] ^+^) molecules, rather than fragment ions. Therefore analysis in the full scan mode (TIC) instead of selected ion mode (SIM), permits the detection and quantification of both known and novel alkaloids in the sample. Two different types of samples were analyzed: *P. microphyllus* leaves and a paste that is left over after the industrial extraction of pilocarpine. The composition of the two samples could be qualitatively compared based on the results of the analysis. A calibration curve based on pilocarpine reference standard was built and used to compare the concentration of imidazole alkaloids in the samples. 

## Results and Discussion

In previous studies of the imidazole alkaloids in *jaborandi*, ESI-MS fingerprinting was used as a fast and simple technique for the comparison of samples [6,7]. However, using direct infusion ESI-MS it was not possible to distinguish between isomers in the plant extracts. The present method was developed and applied in order to chromatographically separate and quantify the alkaloids in the *jaborandi* samples, including the isomers. This method has modified and substantially improved previous methods, such as that proposed by Merbel *et al*. [14], which was isocratic, and separated only four alkaloids in 20 minutes. By using a gradient, we achieved the separation of 13 compounds in 22 minutes. Several of these compounds have only been reported once before in a previous study by our group [7].

**Figure 1:**
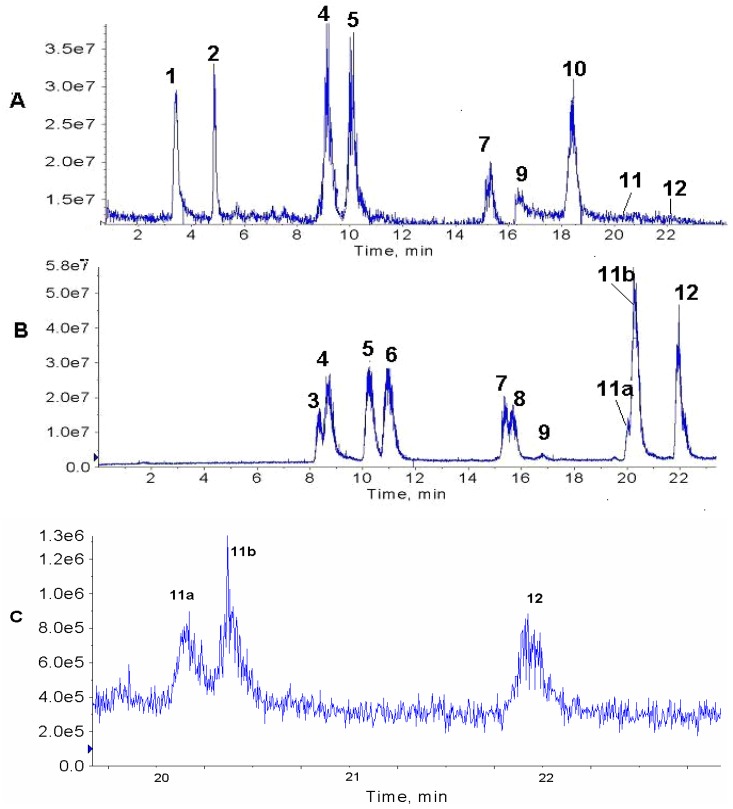
HPLC-MS TIC chromatograms of (A) jaborandi leaf extract and (B) paste extract. Numbered peaks correspond to: **1** *m/z* 202, **2** *m/z* 248, **3** isopilocarpine *m/z* 209, **4** pilocarpine *m/z* 209, **5**, pilosine *m/z* 287, **6** *m/z* 287, **7** *m/z* 287, **8** *m/z* 287, **9** *m/z* 287, **10** *m/z* 316, **11** 3-hydroxymethyl-4-(3methyl-3H-imidazol-4-yl)1-phenyl-butan-1-one *m/z* 259, and **12** anhydropilosine *m/z* 269. (C) XIC of ions *m/z* 259 (**11a** and **11b**) and *m/z* 269 (**12**) from the leaf extract chromatogram.

**Figure 2:**
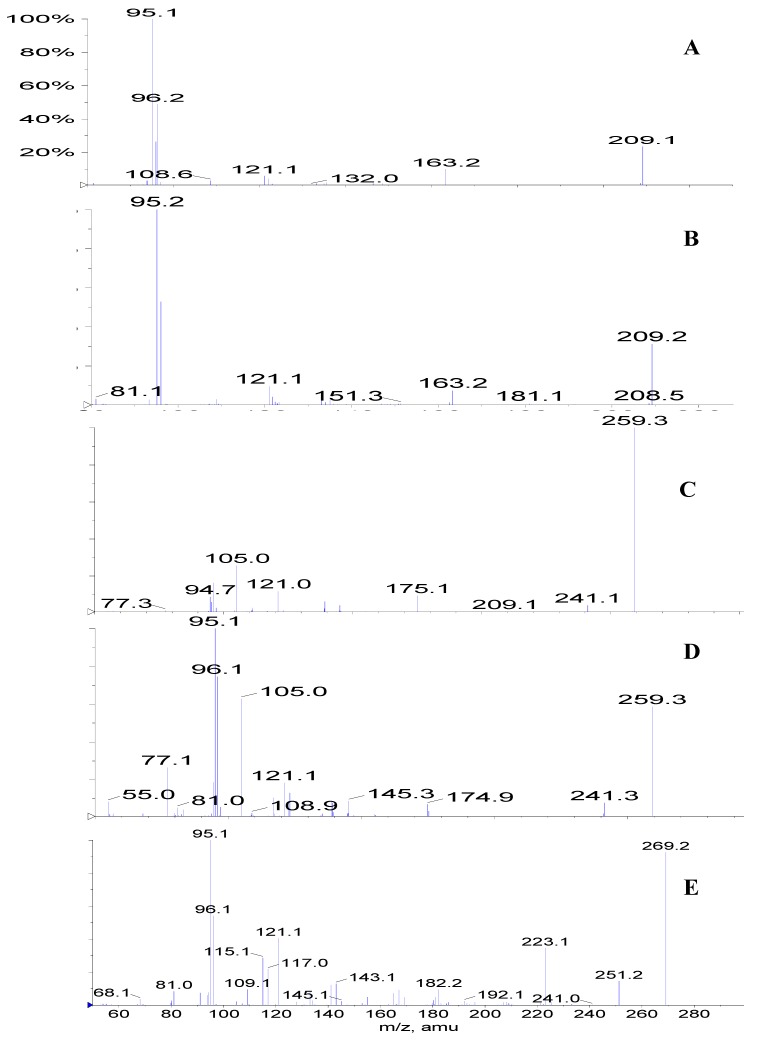

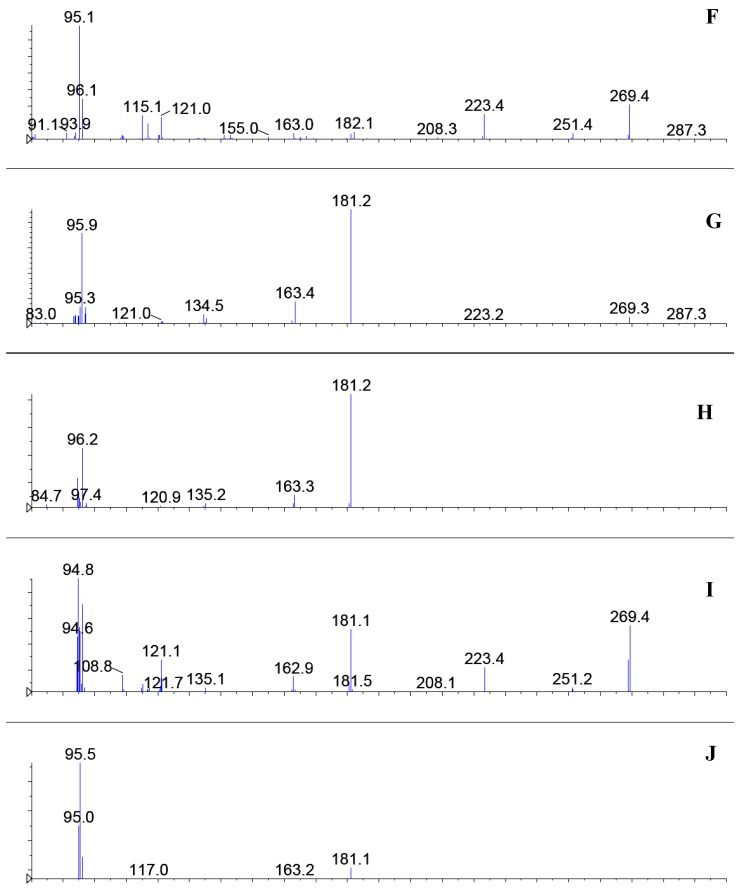
ESI-MS/MS of selected ions from the chromatograms presented in Figure 1: A) *m/z* 209, peak **3**, isopilocarpine; B) *m/z* 209, peak **4**, pilocarpine; C) *m/z* 259, peak **11a**, D) *m/z* 259, peak **11b**; E) *m/z* 269, peak **12**, anhydropilosine; F) *m/z* 287, peak **5**, pilosine, G) *m/z* 287, peak **6**, H) *m/z* 287, peak **7**; I) *m/z* 287, peak **8** and J) *m/z* 287, peak **9**

Figure 1 compares the chromatogram of a leaf extract (A) with that of the paste (B) that is left over after the industrial extraction of pilocarpine. The presence of the epimeric isopilocarpine (**3**) and pilocarpine (**4**) are clearly observed in the paste (Figure 1B), but in the leaf extract only pilocarpine (**4**) is detected. The ESI-MS/MS of the protonated molecules of these isomers of *m/z* 209 are very similar (Figures 2A and B); therefore, they can only be distinguished by their retention times. Both samples were spiked with standard pilocarpine, peak **4** increased in both samples and no new peak appeared in the leaf sample. This confirmed that peak **4** in both chromatograms was pilocarpine. This is in accordance with the results of Merbel *et al*. [14], who reported that isopilocarpine eluted slightly before its isomer, pilocarpine. No other isomers of this molecule have been reported.

A series of peaks corresponding to ions of *m/z* 287 elute (**5, 6, 7, 8** and **9**) in sequence in the paste (Figure 1B), whereas in the leaf extract only **5**, **7** and **9** were observed (Figure 1 A). These compounds have slightly different ESI-MS/MS allowing them to be distinguished, and are presented for the first time in literature in Figure 2 (F, G, H, I and J). We have found references to five isomers of molecular formula C_16_H_18_N_2_O_3_ found in species *P. microphyllus* and *P. jaborandi*: pilosine (2*R*,3*R*,6*S*), isopilosine (2*S*,3*R*,6*R*) and epiisopilosine (2*S*,3*R*,6*S*) [15], as well as piloturin and epiisopiloturin [16] in which the imidazole ring is linked at the C_4_ (rather than at the C_5_ as in pilosine). At the present moment we are able to identify just one of these isomers, that is, pilosine (**5**). Both extracts were spiked with the standard of pilosine to confirm that peak **5** is in fact pilosine, in spite of slight differences in the retention time between samples. Further analysis using a semi-preparative HPLC column should allow us to isolate and identify the other isomers. Isomers were not present in leaves in the same proportion as they were found in the industrial paste. This may be the result of the industrial extraction procedure, as it is known that a long time in the presence of acids (normally used in the extraction of alkaloids) can cause the opening and closing of the lactone ring and formation of isomers. 

Compounds corresponding to peaks **1** (*m/z* 202 ), **2** (*m/z* 248) and **10** (*m/z* 316) in the leaf extract (Figure 1A) have not been identified yet. These ions but have been observed previously in fingerprints of extracts of leaves and callus of *P. microphyllus* [6]. Peaks **11a** and **11b** (*m/z* 259) correspond to a protonated compound identified for the first time in leaves of *P. microphyllus* in a previous study [7], whose suggested structure corresponds to 3-hydroxymethyl-4-(3-methyl-3*H*-imidazol-4-yl)1-phenyl-butan-1-one, based on its ESI-MS/MS. The chromatograms suggest that two isomers are present (Figures 1B and C), although the ESI-MS/MS for both were identical (Figures 2C and D). The observation that the compound of *m/z* 259 is, in fact, a mixture of two isomers, is presented for the first time in this study as a result of the chromatographic method used. Peak **12** (*m/z* 269) corresponds to protonated anhydropilosine, by comparison of its ESI-MS/MS (Figure 2 E) to the compound identified in a previous study [7].

**Scheme 1:**
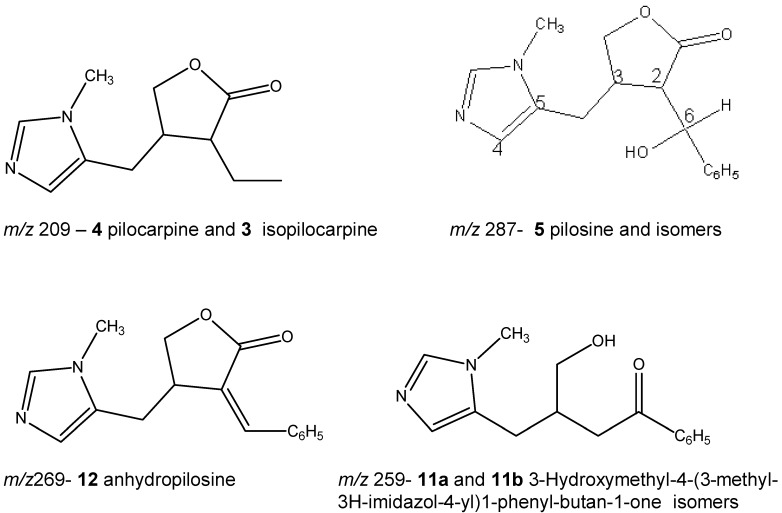


In the present study, chromatograms were obtained using full scan mode (TIC) instead of the selective ion mode (SIM), as the purpose of this study was to observe whichever compounds were present in the extracts rather than observe low concentrations of specific compounds. By using TIC, in a single chromatographic run, several new alkaloids from *Pilocarpus microphyllus*, as well as some previously identified alkaloids could be detected (Figures 1A and B). However, extracting specific ions (XIC) from the chromatogram allows us to observe low concentrations that cannot be seen in the TIC, as exemplified here for *m/z* 259 and 269 in the leaf extract (Figure 1C). Therefore, for the exploratory analysis of samples of alkaloids from *jaborandi*, the TIC mode is the most adequate. However, for the routine analysis of these extracts, using the same chromatographic method in SIM could permit a manifold gain in sensitivity, if such were the need.

The ionization method used by Merbel *et al*. [14] was APCI, which is more energic and causes in-source fragmentation, therefore neither pilocarpic nor isopilocarpic acid could be quantified as parent ions (*m/z* 227), but only as their fragments (*m/*z 209). The use of a much softer ionization (ESI) permitted the observation of all the compounds based on the mass of the protoned [M + H]^+^ molecules, not of fragment ions, which is essential for bio-prospection of new compounds.

A calibration curve with a linear regression coefficient of 0.9949 for values between 0.5 and 20 μg/mL was built using pilocarpine, a value comparable to that obtained by Merbel *et a*l. [14]. This calibration curve enabled us to calculate the concentrations of pilocarpine and isopilocarpine in both extracts (Table 1). It was also possible to obtain an estimate of the concentrations of the other alkaloids based on the area of their peaks in the chromatogram as their areas and concentration fell within the range of the calibration curve, except for compounds **11a, 11b** and **12** in the leaf extract whose concentrations fell below these values. 

**Table 1:** HPLC-ESI-MS analysis of leaf and paste extract (10 μL injections). Areas of peaks and quantification are based on a calibration curve using a pure pilocarpine standard. Compounds are numbered as in Figure 1.

Compound as [M + H]^+^ (*m/z*)	Leaves concentration (µg/ mL)	Paste concentration (µg/ mL)
1 (202)	1.6	
2 (248)	0.9	
3 (209)		1.5
4 (209)	3.0	3.9
5 (287)	2.8	4.1
6 (287)		4.6
7 (287)	1.2	1.9
8 (287)		1.9
9 (287)	0.7	0.2
10 (316)	2.1	
11a (259)		1.2
11b (259)		7.6
12 (269)		4.6

It is noteworthy that the concentration of pilocarpine seems higher in the paste than in the leaf extract. However, one must bear in mind that the paste is enriched of alkaloids since the industrial process removed other leaf components. The relation between the concentrations of pilocarpine in the leaf extract in comparison to the other peaks (24%) is higher than in the paste (12%). Pilosine makes up 22% of the leaf alkaloids but only 14% of the paste. A series of compounds which are found in the paste, but not in the leaf extract, could be degradation products. For example, compounds of *m/z* 259 and 269, whose concentration was below the quantification limit in the leaf extract, made up 42% of the paste. Compounds of *m/z* 202, 248 and 316, observed in the leaf extract, were absent in the paste. These variables could also be linked to the time of the year in which the samples were obtained, as the production of each of these imidazole alkaloids varies, depending on the season [7]. Further studies will be necessary to clarify all these points. However, the estimated concentration obtained using the HPLC-ESI-MS method proposed is sufficient to compare samples from different parts of the plant, of different plants, of plants or callus submitted to different treatments in order to study the production of imidazole alkaloids in this species.

## Conclusions

The HPLC-ESI-MS/MS method for the simultaneous detection and quantification of imidazole alkaloids extracted from *jaborandi* was developed and applied to the comparison of two samples; fresh leaves and the paste left over after industrial pilocarpine extraction. Both samples analyzed contain significant amounts of pilocarpine, as well as a number of other imidazole alkaloids. The alkaloid composition differs between samples qualitatively and quantitatively. Some of the compounds observed in the paste may have been artifacts of the extraction procedure, as they were absent or found in much lower concentrations in the leaf extract. ESI ionization permitted the detection of known and novel alkaloids as their [M + H]^+^ ions, while gradient HPLC permitted the separation of several alkaloids (including isomers). The alkaloid [3-hydroxymethyl-4-(3methyl-3H-imidazol-4-yl)1-phenyl-butan-1-one], observed in a previous study [7], was shown to be a mixture of two isomers of *m/z* 259 through chromatographic separation. On the other hand, three ions (*m/z* 202, 248 and 316) observed previously in ESI-MS fingerprints of leaves and callus of *P. microphyllus* [6] formed individual peaks in the chromatogram. The MS/MS spectra of five isomers of *m/z* 287, pilocarpine, isopilocarpine and three other imidazole alkaloids are reported. 

This method is suitable for the routine analysis of samples containing these alkaloids, as well as for the separation and identification of other alkaloids from this family (bio-prospection) with potential biological and/or pharmacological activity. Several new compounds, which have not been fully characterized yet, may be isolated by preparative HPLC and further investigated by other techniques able to perform detailed structural investigation. The HPLC-ESI-MS/MS method described herein should permit additional studies of the biosynthetic pathway of pilocarpine in *P. microphyllus*. 

## Experimental

### Sample preparation

Leaves of *P. microphyllus* maintained in greenhouse conditions were collected and extracted according to the method of Avancini *et al*. [17], which consists of the following steps: the sample is moistened with 10% NaOH; after 15 min. extraction is carried out three times with CHCl_3_; the pooled organic extracts are re-extracted twice with 2% H_2_SO_4_; the pooled acid extracts are adjusted to pH 12 with NH_4_OH and extracted twice with CHCl_3_. Extraction was carried out immediately prior to analysis from 1 g of the sample. The organic extract was dried in a speed-vac and dissolved in 10 mL of solvent A prior to injection. Solvent A is composed of 0.05 M ammonium acetate buffer adjusted to pH 4 with trifluoroacetic acid [14].

The second material used in this study was the industrial residue (a paste) obtained after leaves of *P. microphyllus* have been used for pilocarpine extraction. This paste was a gift from Centroflora Group (Botucatu, SP, Brazil - http://www.centroflora.com.br) and contains significant levels of pilosine. One g of paste was also extracted as above directly before analysis, dried and dissolved in 10 mL of solvent A prior to injection.

### Standards and solvents

Pure pilocarpine was purchased from Sigma Chemical CO. (St. Louis, MO, USA). Solvents used for the extraction of the leaves of *P. microphyllus* were of analytical grade (Merck, Darmstadt, Germany). Pilosine was a gift from Merck Pharmaceutical Company (Rio de Janeiro, Brazil). For the HPLC-ESI-MS/MS analyses the following solvents and chemicals were used: HPLC grade acetonitrile (Tedia, Fairfield, OH, USA), analytical grade trifluoracetic acid and ammonium acetate (Merck, Darmstadt, Germany) and Milli-Q water (Millipore S.A.S., Molsheim, France).

### HPLC-ESI-MS/MS equipment and conditions

HPLC separation was achieved at ambient temperature (approximately 22-24^o^C) using a Waters Alliance 2695 pump with a 5 μm Inertisil ODS-2 column (250 x 4.6 mm I.D.) obtained from Varian (Middleburg, The Netherlands). A binary gradient was performed starting with 97:3, v/v (solvent A : solvent B) for 8 min (Solvent A - 0.05 M ammonium acetate buffer adjusted to pH 4 with trifluoroacetic acid; Solvent B – acetonitrile) and then ramping to 85:15, v/v (A:B) at 20 min, and held until 23 min. The flow rate was 1 mL/min and a post-column splitter was used to reduce the flow entering the ESI source to approximately 100 μL/min. All ESI-MS and ESI-MS/MS were acquired in the positive ion mode on an Applied Biosystems Q-trap mass spectrometer (Foster City, CA, USA) with a hybrid triple quadrupole/ linear ion trap system, ranging between 100 and 400 *m/*z. The operational parameters used were: capillary 5000 V, temperature 350ºC, declustering potential 70 V and entrance potential 6 V. For the ESI-MS/MS experiments the above conditions were maintained and collision energy was set at 30 V. Nitrogen was used as curtain gas (20 psi), nebulizing gas (20 psi) and collision gas (medium). Injections of 10 μL were used for the samples and standard solutions. The calibration curve was prepared using a stock solution of pilocarpine in water, diluted to concentrations ranging from 0.5 to 20 μg/mL in solvent A prior to injection, as none of the other alkaloids could be found as certified pure standards. Identification of the compounds, when possible, was based on their elution order and MS/MS data in comparison with those from literature. 
